# The STRIPAK complex components FAM40A and FAM40B regulate endothelial cell contractility via ROCKs

**DOI:** 10.1186/s12860-018-0175-y

**Published:** 2018-12-03

**Authors:** Narendra Suryavanshi, Joanna Furmston, Anne J. Ridley

**Affiliations:** 1 Randall Centre for Cell and Molecular Biophysics, King’s College London, New Hunt’s House, Guy’s Campus, London, SE1 1UL UK; 2 School of Cellular and Molecular Medicine, Biomedical Sciences Building, University Walk, University of Bristol, Bristol, BS8 1TD UK

**Keywords:** Cerebral cavernous malformations, Endothelial cells, Actin cytoskeleton, Adherens junctions, Rho/ROCK, STRIPAK complex

## Abstract

**Background:**

Endothelial cells provide a barrier between blood and tissues, which is regulated to allow molecules and cells in out of tissues. Patients with cerebral cavernous malformations (CCM) have dilated leaky blood vessels, especially in the central nervous system. A subset of these patients has loss-of-function mutations in CCM3. CCM3 is part of the STRIPAK protein complex that includes the little-characterized proteins FAM40A and FAM40B.

**Results:**

We show here that FAM40A and FAM40B can interact with CCM3. Knockdown of CCM3, FAM40A or FAM40B in endothelial cells by RNAi causes an increase in stress fibers and a reduction in loop formation in an in vitro angiogenesis assay, which can be reverted by inhibiting the Rho-regulated ROCK kinases. FAM40B depletion also increases endothelial permeability.

**Conclusions:**

These results demonstrate the importance of the FAM40 proteins for endothelial cell physiology, and suggest that they act as part of the CCM3-containing STRIPAK complex.

**Electronic supplementary material:**

The online version of this article (10.1186/s12860-018-0175-y) contains supplementary material, which is available to authorized users.

## Background

Cerebral cavernous malformation is a disease that affects endothelial barrier integrity, particularly in the brain, leading to conglomerations of distended capillaries [1]. Three different genes are mutated in patients with CCM: CCM1/KRIT, CCM2/MGC4607 and CCM3/PDCD10. The three CCM proteins all affect endothelial cell function and have been reported to associate with each other, at least when overexpressed [2], as well as have similar effects on cellular responses, such as autophagy [3]. However, the three proteins have no homology to each other and are found in different signalling complexes [2]. In particular, CCM3 has been identified as a component of the STRIPAK (striatin-interacting phosphatase and kinase) complex, which was identified through an interaction map around the protein phosphatase 2A (PP2A) catalytic subunit by an affinity purification/mass spectrometry approach [4]. This STRIPAK complex includes striatin proteins, which are PP2A regulatory subunit proteins and act as protein scaffolds [5], Mob3, the GCKIII (germinal centre kinase III) kinases (STK24, STK25 and MST4), another germinal centre kinase MINK1 (misshapen like kinase 1) [6], CCM3, and the FAM40 (FAMily with sequence similarity 40) proteins FAM40A/STRIP1 and FAM40B/STRIP2.

CCM3 has been shown to be important for maintaining endothelial junction integrity by preventing stress fiber induction and associated contractility [7]. Mutations in CCM3 lead to vascular malformations in the brain [8]. The GCKIII kinases interact with CCM3 [9], and this interaction is central for CCM3 signalling with GCKIII kinases being downstream effectors of CCM3 function [7]. Wild type CCM3 can bind STK24, STK25 and MST4 while mutant CCM3 protein (with a mutation linked to CCM) fails to do so. Loss of STK25 and CCM3 function leads to vascular development defects in zebrafish and confers a barrier defect in cultured endothelial cells. STK24 and STK25 act downstream of CCM3 to downregulate RhoA activity, preventing loss of barrier function. However, it is not clear if CCM3 signals via RhoA as a second study failed to detect increased RhoA activity upon CCM3 depletion by RNAi [10]. Indeed, vascular anomalies observed upon CCM3 knockdown in zebrafish are distinct from those following either CCM1 or CCM2 knockdown [11]. CCM3 could also contribute to CCM through its role in regulating Golgi positioning and organisation via the GCKIII kinases, thereby regulating endothelial junction formation [12].

Very little is known about the structure or function of the STRIPAK complex-associated FAM40 proteins, which are found in many eukaryotes including fungi. FAM40A and FAM40B have 61% amino acid identity and have two recognised domains, the N-terminal N1221-like domain and the C-terminal DUF3402 (domain of unknown function 3402). Both these domains are functionally uncharacterised and do not resemble any other known protein domains.

The Drosophila FAM40 orthologue was one of many genes identified in a genome-wide RNAi screen for genes affecting cell shape in Drosophila S2 cells [13]. Data from this initial RNAi screen were used to carry out a second RNAi screen in PC3 prostate cancer cells to identify novel regulators of cytoskeletal organisation, cell morphology and migration [14]. Knockdown of FAM40A and FAM40B in PC3 cells resulted in distinct morphological phenotypes: FAM40A-depleted cells had a smaller cell area and high levels of cortical F-actin compared to control cells, whereas FAM40B-depleted cells had a larger spread area and long thin protrusions [14]. Similarly, in A431 cells FAM40A depletion increased actomyosin contractility whereas FAM40B depletion increased cell spreading [15].

Here, we investigate the functions of FAM40A and FAM40B in human endothelial cells, in comparison to CCM3. We find that FAM40A and FAM40B can associate with CCM3, and that all three genes have similar effects in regulating stress fibers and angiogenic loop formation via Rho/ROCK-mediated actomyosin contractility.

## Results

### CCM3 regulates stress fibers and affects endothelial loop formation

**Fig. 1:**
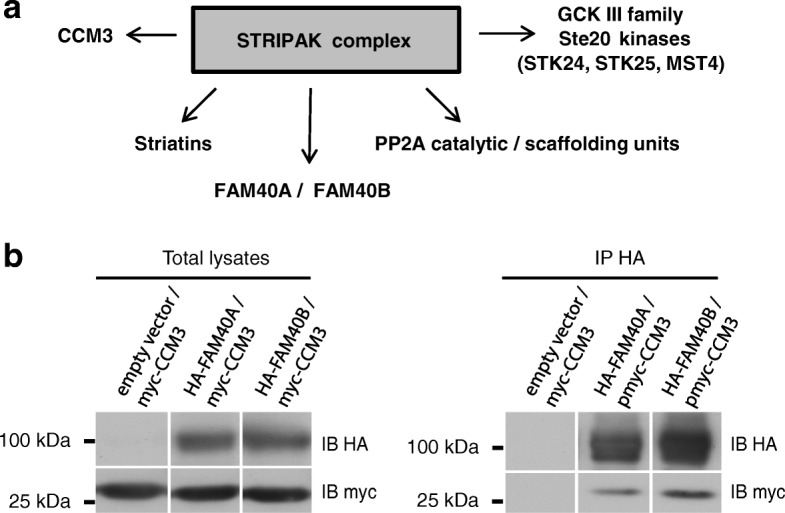
FAM40A and FAM40B interact with CCM3**. a** Schematic showing key components of the human STRIPAK complex. **b** COS7 cells were co-transfected with pDESTHA (empty vector) or pHA-FAM40A or pHA-FAM40B, and pmyc-CCM3. After 48 h, cells were lysed and whole cell lysates incubated with anti-HA epitope agarose beads. Proteins interacting with the HA-FAM40 proteins were visualized by immunoblotting with an antibody to the myc epitope. Lanes are taken from the same immunoblot. Immunoblots are representative of results from 3 independent experiments

The STRIPAK complex contains multiple proteins, including CCM3, FAM40A and FAM40B [4] (Fig. 1a). CCM3 has been reported to bind to GCKIII kinases and striatin [8], but not to FAM40 proteins. We found that FAM40A and FAM40B co-immunoprecipitated with CCM3 when they were co-expressed in COS7 cells (Fig. 1b).

**Fig. 2:**
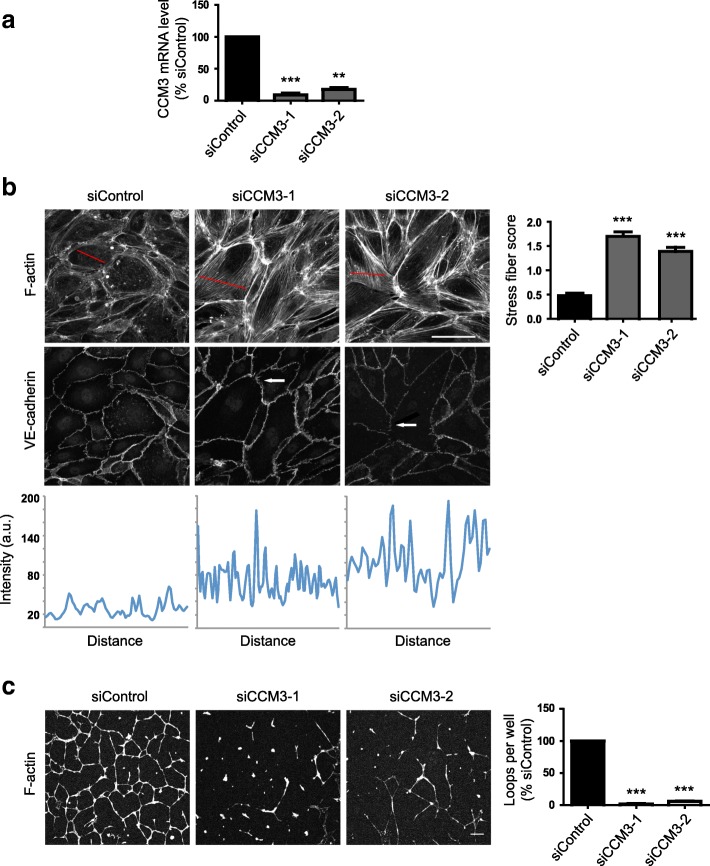
CCM3 regulates stress fibers and angiogenic loop formation in endothelial cells. HUVECs were transfected with siRNAs targeting CCM3 or with a control siRNA. **a** After 72 h, the amount of CCM3 mRNA was determined by qPCR. Data are normalised to GAPDH mRNA levels and are the mean of 3 independent experiments ± SEM. **b** Left panels, HUVECs were seeded onto fibronectin-coated glass coverslips to form confluent monolayers. 72 h after transfection, cells were fixed and stained for F-actin and VE-cadherin. Images are compressed stacks of 10–15 confocal z-sections. Arrows indicate discontinuous junctions (VE-cadherin); Scale bar, 40 μm. Intensity profiles are indicated for representative cells with the region scanned indicated as a red line on each image. Right panel, stress fibers were quantified as described in Materials and Methods; data show mean ± SEM; *n* = 3 independent experiments. At least 150 cells were scored per condition in each experiment. **c** Left panels, 48 h after transfection, HUVECs were seeded onto a layer of Matrigel. Loops were allowed to form for 24 h, and then cells were fixed and stained for F-actin with phalloidin-Alexa546. Scale bar, 100 μm. Right, loop formation was quantified by scoring the number of loops per field using fluorescence images. 6 fields were scored per condition in each experiment. Results are shown as % of siControl. Data are mean ± SEM, n = 3 independent experiments; **p < 0.01, ***p < 0.001 compared to siControl determined by Student’s t-test

The CCM proteins have been shown to be important for maintaining endothelial homeostasis by regulating cell contractility. Depletion of CCM3 was reported to lead to an induction of stress fibers in endothelial cells [7]. We used two independent siRNAs targeting CCM3, which consistently depleted CCM3 mRNA by at least 85% (Fig. 2a). Human umbilical vein endothelial cells (HUVECs) depleted of CCM3 exhibited increased stress fibers (Fig. 2b). VE-cadherin localization at cell-cell junctions was discontinuous along junctions at the ends of cells where stress fibers from neighbouring cells met at junctions (Fig. 2b; arrows), similar to our previous observations in TNFα-stimulated HUVECs [16]. We tested the effect of CCM3 depletion in an in vitro loop formation angiogenesis assay on Matrigel [17]. CCM3-depleted HUVECs had strong defects in endothelial loop formation (Fig. 2c).

### FAM40A and FAM40B regulate stress fibers in endothelial cells

**Fig. 3:**
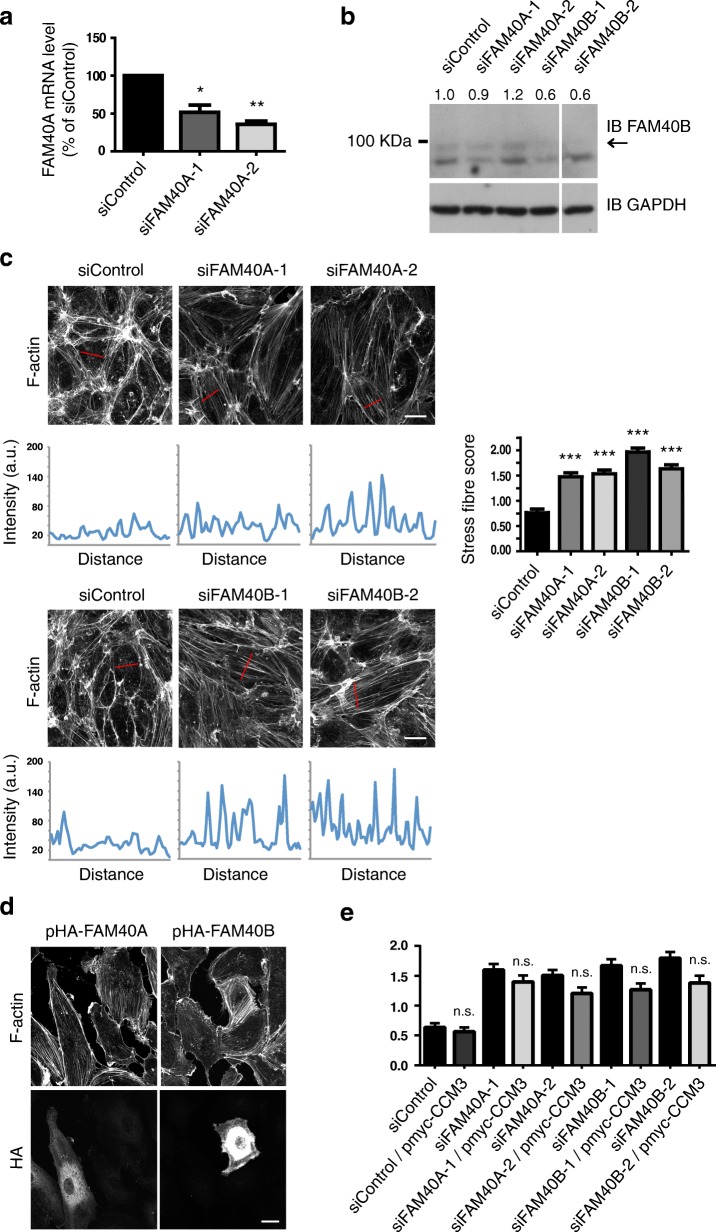
FAM40A and FAM40B regulate stress fibers in endothelial cells. HUVECs were transfected with siRNAs targeting FAM40A, FAM40B or with a control siRNA, and analysed 72 after transfection. **a** The amount of FAM40A mRNA was determined by quantitative PCR. Data are normalised to GAPDH mRNA levels and are the mean ± SEM; *n* = 3. **b** Representative immunoblot of HUVEC lysates. Whole cell lysates were immunoblotted for FAM40B and GAPDH as a loading control; arrow indicates FAM40B protein. Lanes are from the same immunoblot. Quantification of FAM40B band intensities (to band) is shown above the blot, relative to siControl, and was carried out using ImageJ. **c** Cells were seeded onto fibronectin-coated glass coverslips to form confluent monolayers. Cells were fixed and stained for F-actin. Images are compressed stacks of 10–15 z-sections. Scale bar, 40 μm. Intensity profiles are indicated for representative cells with the region scanned indicated as a red line on each image. Graph (right panel) shows quantification of stress fibers from at least 150 cells per condition from 3 independent experiments ± SEM. **d** HUVECs were transfected with pHA-FAM40A, pHA-FAM40B or pmyc-CCM3, seeded onto fibronectin-coated glass coverslips and fixed after 24 h. Cells were stained for F-actin and the HA or myc epitope. Images are compressed stacks of 15–20 z-sections (FAM40A/B) or single images (CCM3) and are representative of 2 independent experiments. Scale bar, 20 μm. **e** HUVECs were transfected with control siRNA or siRNAs targeting FAM40A or FAM40B, followed after 48 h by transfection with pmyc-CCM3. After a further 24 h, stress fibers were quantified from at least 100 cells per condition from 2 independent experiments. *p < 0.05, **p < 0.01, ***p < 0.001, n.s. not significant; Student’s *t*-test, compared to siControl

Given the strong phenotype of CCM3 depletion in endothelial cells, we investigated whether the CCM3-interacting proteins FAM40A or FAM40B induced similar phenotypes. Two independent siRNAs targeting FAM40A reduced FAM40A mRNA levels by at least 50% in HUVECs (Fig. 3a). No antibody specific to FAM40A is available, but an antibody to FAM40B showed that two siRNAs targeting FAM40B reduced protein expression levels (Fig. 3b). Depletion of FAM40A or FAM40B in HUVECs induced an increase in stress fibers (Fig. 3c).

Since both FAM40 depletion and CCM3 depletion induced an increase in stress fibers, we wondered whether CCM3 overexpression could reduce the response to FAM40 depletion. However, CCM3 overexpression did not rescue the FAM40 depletion-induced stress fiber response (Fig. 3d), indicating that FAM40 proteins are not directly upstream of CCM3. Instead, they probably all act together in the STRIPAK complex to regulate stress fiber assembly. This is consistent with our observations with FAM40A/B overexpression (Fig. 3e). Neither induced a decrease in stress fibers, consistent with a model in which multiple components of the complex act together to regulate stress fiber levels. Both FAM40A and FAM40B showed a diffuse cytoplasmic localization in HUVECs, similar to CCM3 (Fig. 3e).

### Rho/ROCK signalling is required for FAM40-regulated stress fibers

**Fig. 4:**
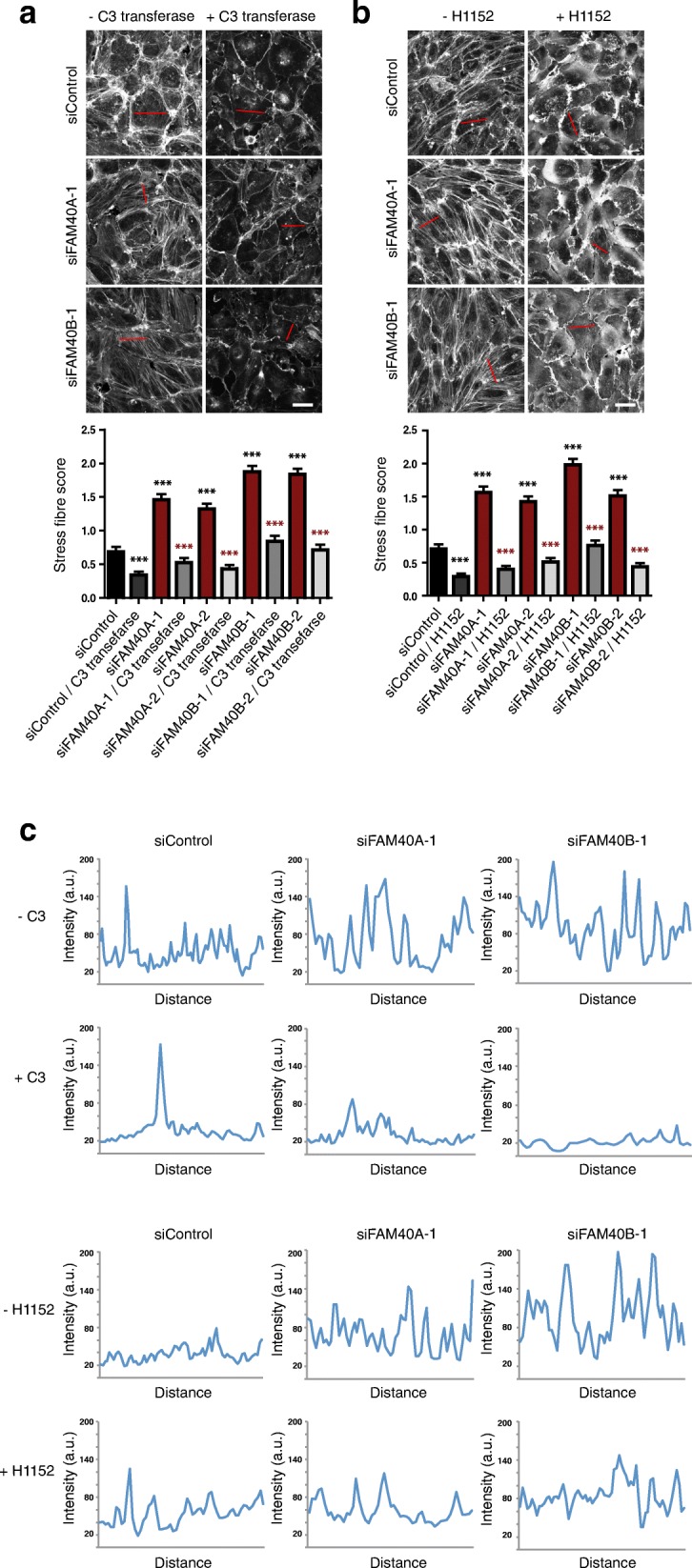
Rho/ROCK signaling is required for stress fiber induction by FAM40 depletion. HUVECs were transfected with siRNAs targeting FAM40A, FAM40B or a control siRNA and seeded onto fibronectin-coated glass coverslips to form confluent monolayers. 72 h after transfection cells were treated with (**a**) 4 μg/ml C3 transferase for 2 h, or (**b**) 5 μM H1152 for 10 min after which they were fixed and stained for F-actin. Images are compressed stacks of 10–15 z-sections. Scale bars, 40 μm. Graphs show stress fiber content quantified from at least 150 cells per condition from 3 independent experiments. Error bars depict SEM values. *** (black) *p* < 0.001; Student’s *t*-test, compared to siControl; *** (grey) *p* < 0.001; Student’s *t*-test, comparison between siRNA+/− C3 transferase or H1152. (**c**) Intensity profiles are indicated for representative cells with the region scanned indicated as a red line in each image in panels 4A and 4B

Given that stress fiber formation is generally regulated by Rho family GTPases, particularly the Rho subfamily members RhoA, RhoB and RhoC [18], we tested whether these Rho subfamily proteins were required for the increase in stress fibers in response to FAM40A or FAM40B depletion. HUVECs were incubated with the Clostridium botulinum exoenzyme C3 transferase, which ADP-ribosylates RhoA, RhoB and RhoC and thereby inhibits their activity [19]. C3 transferase prevented the increase in stress fibers induced by FAM40A or FAM40B depletion (Fig. 4a). It also reduced the level of non-stress fiber actin filaments (as observed in siControl-transfected cells).

Stress fiber formation downstream of Rho subfamily proteins is dependent on their downstream targets ROCK1 and ROCK2 [20]. A small molecule inhibitor of ROCK1/2 kinase activity, H1152 [21], also prevented the increase in stress fibers induced by FAM40A or FAM40B depletion (Fig. 4b). These results indicate that FAM40A and FAM40B normally suppress Rho/ROCK-induced stress fiber assembly. Although H1152 is highly selective for ROCKs, it can inhibit other kinases at higher concentrations in vitro [28], and hence it is possible that some of its effects are due to other kinases.

**Fig. 5:**
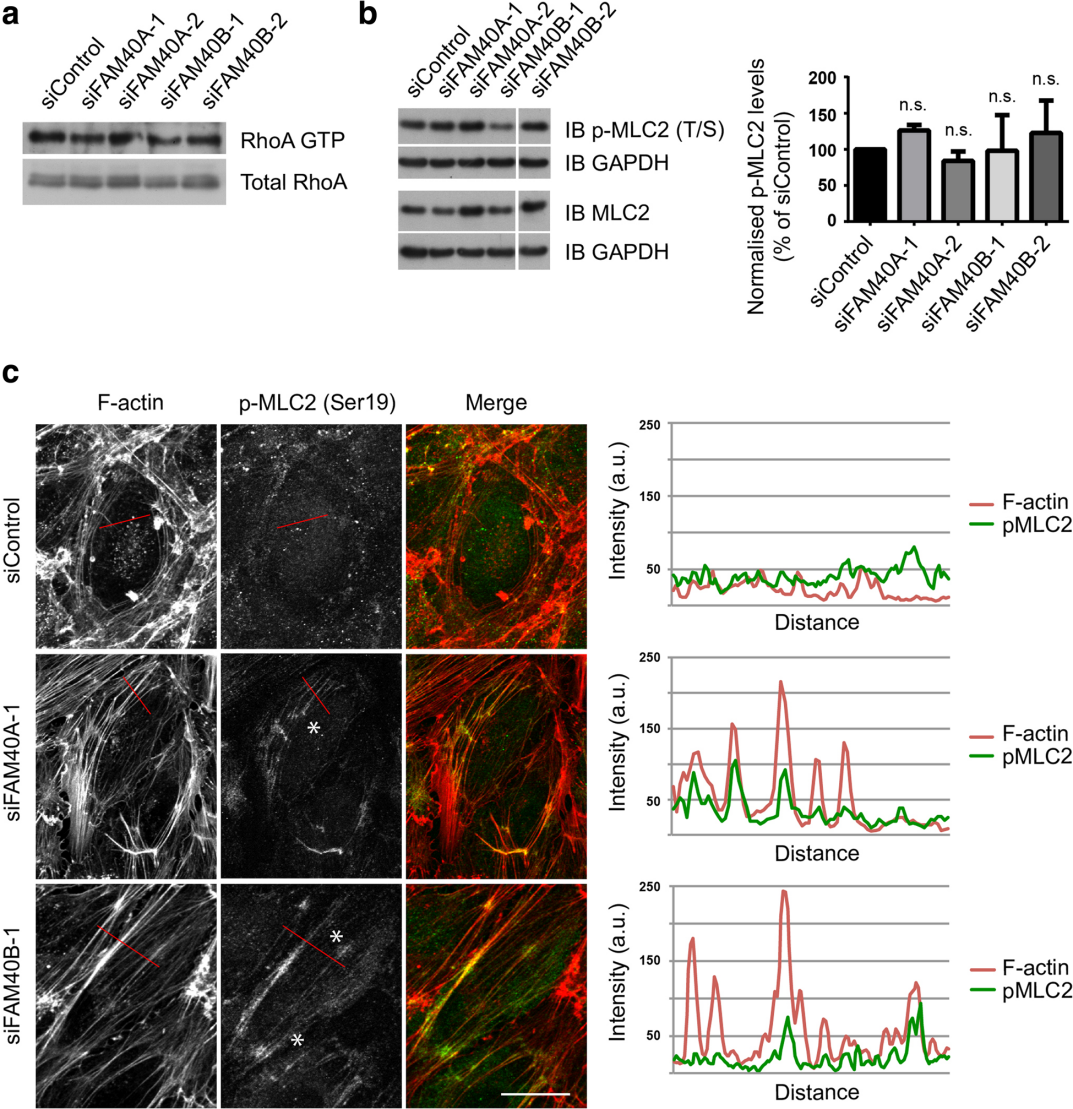
Effects of FAM40A or FAM40B on RhoA activity and MLC2 phosphorylation. **a** HUVECs were transfected with siRNAs targeting FAM40A or FAM40B. After 72 h, cells were lysed and whole cell lysates used in a GST-RBD pulldown assay to determine levels of active GTP-loaded RhoA. A representative immunoblot for total and GTP-loaded RhoA is shown (*n* = 3). **b** Immunoblot of phospho-MLC2 (pMLC2) and MLC2 in FAM40A and FAM40B-depleted HUVECs. Quantification of pMLC2 levels was performed by individually normalising pMLC2 and MLC2 levels to GAPDH levels. The ratio of the normalised pMLC2 level to the normalised MLC2 level was used as a measure of MLC2 phosphorylation. Data are means of 3 independent experiments ± SEM. n.s. not significant; Student’s *t*-test compared to siControl. **c** HUVECs were transfected with siRNAs targeting FAM40A or FAM40B and seeded onto fibronectin-coated glass coverslips to form confluent monolayers. 72 h after transfection cells were fixed and stained for F-actin and pMLC2. Images are compressed stacks of 10–15 z-sections. Asterisks indicate examples of regions where pMLC2 co-localizes with stress fibers. Images shown are compressed stacks of 10–15 z-sections and are representative of 2 independent experiments. Scale bar, 20 μm. Dual colour intensity profiles for F-actin and pMLC2 are indicated for representative cells with the region scanned indicated as a red line on each image

We did not observe an increase in total RhoA activity in FAM40-depleted cells (Fig. 5a), or levels of phosphorylated MLC2 (pMLC2) (Fig. 5b), which is often considered a marker for ROCK activity [20]. As a control, thrombin stimulation increased pMLC levels in HUVECs (Additional file 1: Figure S1), as previously reported [22]. Notably, pMLC2 localized along stress fibers in FAM40-depleted HUVECs (Fig. 5c), indicating that they are contractile.

### FAM40A and FAM40B depletion affects endothelial junctions and permeability

**Fig. 6:**
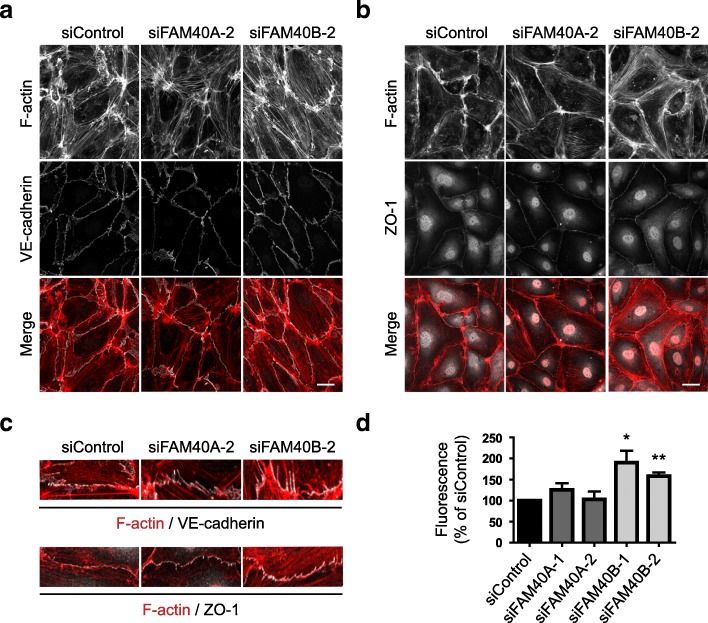
Effect of FAM40A and FAM40B depletion on endothelial junctions and permeability. **a** HUVECs were transfected with siRNAs targeting FAM40A or FAM40B and seeded onto fibronectin-coated glass coverslips to form monolayers. 72 h after transfection cells were fixed and stained for F-actin and VE-cadherin. **b** Immunofluorescence analysis of FAM40A and FAM40B-depleted HUVECs for F-actin and ZO-1. Images are compressed stacks of 10–15 z-sections and are representative of 3 independent experiments. Scale bars, 40 μm. c Magnified images highlighting anchoring of stress fibers at cell-cell junctions. **d** HUVECs were transfected with the indicated siRNAs and plated at confluency onto transwell inserts. 72 h after transfection, FITC-dextran was added to the upper chamber. After a further 80 min the fluorescence of media in the lower chamber was determined. Data show mean permeability ± SEM as % of siControl; *n* = 3 independent experiments for siFAM40A; *n* = 5 independent experiments for siFAM40B. *p < 0.05, **p < 0.01; Student’s *t*-test, compared to siControl

We have previously shown that stress fibers in endothelial cells are connected to cell-cell junctions and affect junctional integrity [16]. We therefore investigated whether FAM40A or FAM40B altered the distribution of adherens junctional or tight junctional proteins. Neither VE-cadherin (Fig. 6a) nor ZO-1, a component of tight junctions (Fig. 6b) disappeared from junctions following FAM40A or FAM40B depletion. However, in FAM40-depleted cells, VE-cadherin localization at cell-cell junctions was discontinuous along junctions at the ends of cells where stress fibers from neighbouring cells met at junctions (Fig. 6c), similar to our observations with CCM3 depletion (Fig. 2b). Moreover, in FAM40B-depleted cells, endothelial permeability was increased (Fig. 6d), consistent with disruption in cell-cell junctions. FAM40A depletion did not affect endothelial permeability, suggesting that FAM40A and FAM40B could mediate different as well as common functions in endothelial cells.

### FAM40A and FAM40B affect endothelial loop formation

**Fig. 7:**
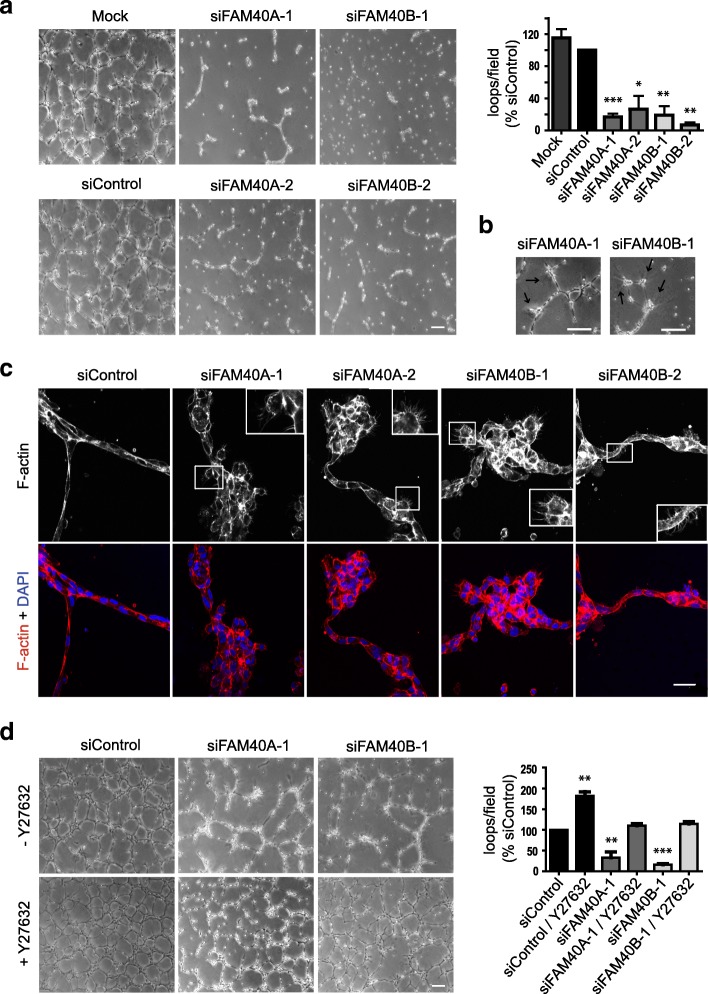
FAM40A and FAM40B regulate endothelial loop formation. **a** HUVECs were transfected with siRNAs targeting FAM40A and FAM40B. 48 h after transfection cells were seeded onto a layer of Matrigel. Loops were allowed to form for 24 h. Scale bar, 200 μm. Graph (right panel) shows quantification of number of loops per field. At least 5 fields were scored per condition in each experiment. Data show mean ± SEM normalised to siControl; *n* = 4 for siFAM40A-1, siFAM40A-2 and siFAM40B-1; *n* = 3 for siFAM40B-2. **b** Arrows highlight deformation of the Matrigel substrate by FAM40-depleted cells. Scale bars, 200 μm. **c** HUVECs were allowed to form loops on polymerised Matrigel for 24 h, and were fixed and stained for F-actin, and with DAPI to visualise nuclei. Images are sections taken at a single z-position and are representative of 3 independent experiments. Boxed areas are shown at higher magnification (inset images) to highlight F-actin micro-spikes on cells. Scale bar, 40 μm. **d** FAM40A and FAM40B-depleted HUVECs were allowed to form loops on polymerised Martigel for 24 h. Cells were treated with 10 μM Y-27632 for 24 h. Scale bar, 200 μm. Graph (right panel) shows quantification of number of loops per field. At least 4 fields were scored per condition in each experiment. Data are mean ± SEM normalised to siControl, *n* = 3 independent experiments. *p < 0.05, **p < 0.01, ***p < 0.001; Student’s *t*-test, compared to siControl


Our results with CCM3 indicate that increased stress fibers correlate with reduced endothelial loop formation (Fig. 2). We therefore tested the effects of FAM40A and FAM40B in this assay. Knockdown of both FAM40A and FAM40B results in strong defects in the loop formation angiogenesis assay (Fig. 7a). Indeed, very few loops were observed. Movies showed that the FAM40-depleted endothelial cells transiently extended protrusions but then collapsed into cell groups (Fig. 7b, Additional files 2, 3, 4, 5, 6, and 7). Analysis of F-actin distribution indicated that control cells formed chains of single cells along the loops, whereas FAM40A or FAM40B-depleted cells were predominantly in clumps (Fig. 7c). Cells at the edge of the clumps had multiple thin protrusions that are likely to be retraction fibers which form as cells pull back from the matrix.

**Additional file 2: siControl**. (AVI 1920 kb)



**Additional file 3: siControl+Y**. (AVI 1820 kb)



**Additional file 4: siFAM40A**. (AVI 2030 kb)



**Additional file 5: siFAM40A+Y**. (AVI 2490 kb)



**Additional file 6: siFAM40B**. (AVI 1680 kb)



**Additional file 7: siFAM40B+Y**. (AVI 1860 kb)


Given that inhibition of ROCK activity reduced stress fiber levels in FAM40-depleted cells, we tested whether ROCK also affected loop formation. Treatment with the ROCK inhibitor Y-27632 rescued FAM40 depletion-induced inhibition of loop formation (Fig. 7d, Additional file 2). Taken together, these results indicate that ROCK-mediated contractility drives the inhibition of loop formation in FAM40-depleted cells.

## Discussion

CCM3 associates with the STRIPAK complex, which includes kinases and phosphatases and scaffolding proteins [5]. CCM3 is well known to affect endothelial cell function in vitro and in vivo [2, 8]. FAM40A and FAM40B are little-characterized members of the STRIPAK complex, and their function in endothelial cells has not previously been addressed. Here, we show that CCM3 co-immunoprecipitates with FAM40A and FAM40B when exogenously expressed in COS7 cells, indicating a close association between the proteins. We demonstrate that FAM40A and FAM40B depletion, like CCM3 depletion, increases stress fibers and junctional disruption in endothelial cells, and impairs angiogenesis in a loop formation assay.

In endothelial cells, we found that depletion of either FAM40A or FAM40B increased stress fibers in endothelial monolayers, and cell contractility in loop angiogenesis assays. In contrast, in PC3 cells, MDA-MB-231 breast cancer cells and A431 cells, FAM40A and FAM40B depletion leads to distinct phenotypes. For example, FAM40A depletion resulted in reduced cell spreading, increased membrane blebbing and higher levels of staining for phosphorylated MLC2 and phosphorylated ezrin/radixin/moesin proteins (pERM), whereas FAM40B depletion increased cell spreading [14, 15]. While depletion of FAM40A and STRN3 gave similar phenotypes, depletion of other STRIPAK complex components, the GCKIII kinases MST3 and MST4, led to a phenotype that resembled FAM40B depletion [15]. This suggests that different components of STRIPAK complexes have opposing functions, and thus it is possible that distinct subcomplexes exist dynamically in cells to regulate cell shape and the cytoskeleton.

Whether FAM40A and FAM40B have different functions or act in distinct complexes in endothelial cells is not known. While their depletion had similar phenotypes in nearly all the assays we used, only FAM40B affected endothelial permeability, suggesting it might have a unique function at endothelial junctions. Alternatively, it could be that FAM40B is expressed at higher levels than FAM40A in HUVECs and thus has a stronger effect on permeability. It would be interesting to compare the effects of FAM40A and FAM40B depletion in other types of endothelial cells, such as brain endothelial cells which have better-developed tight junctions than HUVECs [1].

It is still unclear how CCM3 and other members of the STRIPAK complex regulate actomyosin contractility and cell shape. Although it has been reported that depletion of CCM1 and CCM2 increases RhoA activity in human endothelial cells [23], we and others have not observed any change in RhoA activity following CCM3, FAM40A or FAM40B depletion [15], even though Rho and ROCK function is required for the stress fiber formation we observe in FAM40A/B-depleted endothelial cells, and phospho-MLC2 staining is present on the stress fibers. Instead, it has been suggested that CCM3 regulates the localisation of the STRIPAK complex GCKIII kinases MST3 and MST4 under some conditions [15], although it is not known whether FAM40A or FAM40B have a similar function.

Given their effects on cell morphology and the actin cytoskeleton, FAM40 proteins would be expected to influence cell migration. Indeed, the single FAM40 gene in Drosophila is required for optimal border cell migration in the Drosophila egg chamber [15]. Similarly, both FAM40A and FAM40B depletion slightly reduces PC3 cell migration in 2D [14]. However, in MDA-MB-231 cells, FAM40B depletion increases migration in 2D, although it reduces migration through small pores [15]. In endothelial cells, we did not observe any effects of FAM40A or FAM40B on migration in a modified scratch wound assay (unpublished data). These differences in the effects of FAM40A and FAM40B on 2D migration may reflect different cell types or conditions, and the fact that FAM40 proteins do not appear to influence Rho GTPase activity directly.

In addition to the well-characterized roles of CCM proteins in endothelial cells, there is increasing evidence that they contribute to the functions of other cell types, including neurons and cancer cells [5]. For example, CCM3 facilitates interactions between neuroglia and endothelial cells [24], while FAM40B has been shown to be important for maintaining cell-cell contacts in HeLa cells [14]. FAM40B and the STRIPAK complex components CCM3 and the GCKIII kinases MST3 and MST4 are all required for optimal survival of MDA-MB-231 breast cancer cells in the lungs in vivo [15], although whether this reflects their roles in cell migration or attachment to endothelial cells is not known. The Far complex in budding yeast is related to the human STRIPAK complex, and has been proposed to facilitate the fusion of mating cells [25]. These results together with our data in endothelial cells suggest that a general role of the FAM40 proteins/STRIPAK complex could be in mediating cell-cell contact formation and/or maintenance.

FAM40A and FAM40B do not have any known catalytic function, and are more likely to be scaffolding proteins that facilitate protein-protein interactions, although the only known direct interacting partners are striatins [4]. Striatins themselves are also considered as protein scaffolds, and indeed act in several different protein complexes to facilitate signaling [26]. Interestingly, depletion of the striatin STRN3 in human A431 carcinoma cells led to a similar phenotype to FAM40A depletion, indicating that they act on the same pathway.

## Conclusions

Overall, our observations that CCM3, FAM40A and FAM40B depletion all induce a similar phenotype in endothelial cells imply that CCM3 acts via the STRIPAK complex to regulate endothelial function, and thus that altering the activity of specific STRIPAK components could alleviate the symptoms in patients with cerebral cavernous malformation.

## Methods

### Antibodies

The following antibodies were used for western blotting: rat anti-HA (3F10; Roche/Sigma-Aldrich), rabbit anti-myc, mouse anti-RhoA, rabbit anti-MLC2 (Santa Cruz Technology), rabbit anti-FAM40B (Sigma-Aldrich), rabbit anti-pThr18/pSer19-MLC2 (Cell Signaling), mouse anti-GADPH (Millipore), secondary HRP-conjugated sheep anti-mouse IgG and donkey anti-rabbit IgG (GE Healthcare). Antibodies for immunofluorescence analysis were: rabbit anti-HA (Santa Cruz Technology), rabbit anti-pSer19-MLC2 (Cell Signaling), mouse anti-VE-cadherin (BD Biosciences), rabbit anti-ZO-1 (#61–7300; ThermoFisher Scientific), secondary AlexaFluor-488 and -647-conjugated antibodies (Molecular Probes). Cells were also stained with DAPI (DNA) and AlexaFluor-546-labeled phalloidin (F-actin; Molecular Probes).

### Cell culture and siRNA transfection

Pooled human umbilical vein endothelial cells (HUVECs) obtained from Lonza or PromoCell were cultured in EBM-2 medium containing the appropriate growth factors (EGM-2) and supplemented with 2% foetal bovine serum (FBS). Cell culture dishes were coated with 10 μg/ml fibronectin for 1 h at 37 °C prior to cell seeding. HUVECs were used until passage 4. Where indicated, they were stimulated with 1 U/ml thrombin (Sigma-Aldrich) for 10 min.

COS7 cells were cultured in DMEM containing 10% FBS, penicillin (100 IU/ml) and streptomycin (100 μg/ml).

HUVECs were transfected with 50 nM siRNAs using Oligofectamine in EGM-2 medium containing 4% FCS. After 16 h, medium was changed to EGM-2 containing 2% FCS. Cells were analysed 72 h after siRNA transfection. The following siRNA sequences were obtained from Dharmacon (GE Healthcare): siFAM40A-1 (5’-GCUGAUGACUCUCGAGAAG-3′), siFAM40A-2 (5’-CAGCACAAGUACACGUCGA-3′), siFAM40B-1 (5’-GAAGGCAACUCCUCACUAA-3′), siFAM40B-2 (5’-UGACUGGGCUUACGGGAAU-3′), siControl (5’-UGGUUUACAUGUCGACUAA-3′). CCM3 siRNAs were obtained from Ambion (ThermoFisher Scientific): siCCM3–1 (5’-GUGAUACUCUGAAAACGUA-3′), siCCM3–2 (5’-AGAAAAUCCAGGUCUCACA-3′).

### cDNA cloning and plasmid transfection

Human FAM40A, FAM40B and CCM3 cDNAs were kindly provided in pENTRY by Dr. Stefan Wiemann (DKFZ, Germany). The FAM40A and FAM40B cDNAs were cloned into pDEST-HA, and CCM3 into pDEST-myc, using a Gateway™ LR Clonase II Enzyme kit (Invitrogen) as recommended by the manufacturer.

HUVECs were transfected with plasmid DNA with an Amaxa Nucleofector (Lonza) according to the manufacturer’s instructions. After nucleofection, cells were plated onto fibronectin-coated glass coverslips for immunofluorescence analysis.

COS7 cells were transfected with plasmid DNA using a Genepulser II System (Bio-Rad) at 250 V, 975 μF in electroporation buffer (120 mM KCl, 10 mM K_2_PO_4_/KHPO_4_ pH 7.6, 25 mM HEPES pH 7.6, 2 mM MgC_l2_, 0.5% Ficoll 400). After 48 h, cells were lysed for immunoprecipitation analysis.

### Immunoprecipitation and immunoblotting

Cells were lysed in either IP lysis buffer (20 mM Tris-Cl pH 8, 130 mM NaCl, 1% Triton X-100, 1 mM DTT, 10 mM NaF, EDTA-free protease inhibitor cocktail (Roche Applied Science), phosphatase inhibitor cocktail (Calbiochem)) or RIPA buffer (20 mM Tris-Cl pH 7.5, 150 mM NaCl, 1 mM EDTA, 1% NP-40, 0.5% sodium deoxycholate, 0.1% SDS, 1 mM DTT, 125 mM NaF, EDTA-free protease inhibitor cocktail, phosphatase inhibitor cocktail). For immunoprecipitations, COS7 cell lysates were pre-cleared by incubation with non-immune IgG agarose beads (Sigma-Aldrich) for 1 h at 4 °C, then incubated with mouse anti-HA agarose beads (Sigma-Aldrich) for 3 h at 4 °C. Beads were washed with high salt IP lysis buffer (containing 250 mM NaCl). Proteins were eluted from the beads with sample buffer (4% SDS, 160 mM Tris-Cl pH 6.8, 20% glycerol, 10 mM DTT). Empty vector was transfected as a control to demonstrate that the anti-HA beads did not pull down myc-CCM3 directly.

For pMLC2 analysis, HUVECs were lysed with Laemmli lysis buffer (80 mM Tris-Cl pH 7.5, 10% glycerol, 2% SDS, 10 mM glycerol phosphate, 1 mM Na_3_VO_4_, 1 mM DTT, 10 mM NaF, 1 mM PMSF, EDTA-free protease inhibitor cocktail). Cell lysates were snap frozen in liquid nitrogen, then boiled for 5 min and sonicated before centrifugation.

Cell lysates were separate by SDS-PAGE using pre-cast 4–12% Bis-Tris gels (Life Technologies). Proteins were transferred onto PVDF membranes (Immobilon-P; Millipore). Membranes were blocked in 5% skimmed milk powder 5% BSA, incubated with primary antibodies in blocking solution, followed by HRP-conjugated secondary antibodies and detection by enhanced chemiluminescence (ECL) (GE Healthcare) according to the manufacturer’s instructions. Bands on immunoblots were quantified by densitometric analysis using ImageJ software.

### RhoA activity assay

GST-Rhotekin-RBD was purified from *E. coli* on glutathione sepharose beads (GE Healthcare) as previously described [27]. HUVECs were lysed with Rho lysis buffer (50 mM Tris-Cl pH 7.5, 500 mM NaCl, 10 mM MgCl_2_, 10% glycerol, 0.1% SDS, 1% Triton X-100, 0.5% sodium deoxycholate, 25 mM NaF, 1 mM Na_3_VO_4_, 1 mM PMSF, EDTA-free protease inhibitor cocktail). A small aliquot of the lysate was kept to determine total RhoA levels. Lysates were then incubated with GST-RBD for 1 h at 4 °C with rotation. Protein was eluted from the beads by boiling with 4× Laemmli sample buffer and analysed by western blotting.

### Immunofluorescence and confocal microscopy

HUVECs were seeded onto glass coverslips coated with fibronectin (10 μg/ml at 37 °C for overnight). Cells were fixed in 4% paraformaldehyde, permeabilized with 0.1% Triton X-100 and blocked in 3% BSA. Primary antibodies were diluted in 1% BSA in PBS. Fluorophore-conjugated secondary antibodies, DAPI and phalloidin were prepared in the same way as the primary antibodies. Coverslips were mounted onto glass slides using fluorescent mounting medium (DAKO).

A Zeiss LSM510 confocal laser-scanning microscope with an EC Plan-Neofluar 40×/1.30 Oil DIC M27 or a Plan-Apochromat 63×/1.40 Oil DIC M27, and ZEN software was used to take images of fluorescently stained cells. Images in each experiment were acquired using the same gain and offset settings.

Stress fibers were quantified by assigning a score to each cell based on the stress fiber content in the centre of the cell; 0 – few or no stress fibers, 1 – up to 50% of the cell centre contains stress fibers, 2–50% to 75% of the cell centre contains stress fibers, 3 – greater than 75% of the cell centre contains stress fibers. The experimenter quantifying stress fibers was blinded to the treatment.

### Endothelial permeability assay

HUVECs were transfected with siRNAs and after 48 h were plated onto fibronectin-coated (10 μg/ml at 37 °C for 1 h) Transwell filters (12-mm diameter, 0.4-μm pore size, Costar) to form confluent monolayers. After 24 h, 0.1 mg/ml FITC dextran (molecular weight 42 kDa) was added to the upper chamber. Fluorescence was measured in the lower chamber after 80 min using a microplate analyser (Fusion-FA; PerkinElmer; excitation, 485 nm; detection, 523–535 nm). Each condition was performed in triplicate.

### Angiogenic loop formation assay

Matrigel (BD Biosciences, at least 9 mg/ml) was diluted 1:1 with PBS, 300 μl added to each well of a 6-well dish and allowed to polymerize for 1.5 h. HUVECs were transfected with siRNAs and after 48 h 2 × 10^5^ cells per well were seeded onto Matrigel, with or without addition of 10 μM ROCK inhibitor Y-27632 (Calbiochem). Cells were imaged after 24 h by phase-contrast microscopy using a Nikon TE2000-E microscope with a Plan Fluor 4× or 10× objective (Nikon) and a Hamamatsu Orca-ER digital camera, or fixed, permeabilized and stained for F-actin as described above (Immunofluorescence and confocal microscopy). The number of loops formed per imaged field was counted. The mean value of loops for multiple fields was used for statistical analysis. Alternatively, phase-contrast time-lapse movies were acquired 1 h after seeding cells onto Matrigel at 37 °C and 5% CO_2_. Images were captured using Metamorph software at a frame rate of 1 frame/30 min for 24 h.

### qPCR

Total RNA was extracted 72 h after siRNA transfection using either an RNeasy mini kit (Qiagen) according to the manufacturer’s instructions or with Trizol and chloroform extraction. Purified RNA was treated with DNase (DNA free kit, Ambion). A SuperScript® VILO™ cDNA synthesis kit (Invitrogen) was used to synthesise cDNA according to the manufacturer’s instructions.

qPCR was carried out with SYBR green detection chemistry (mastermix from Primer Design). GAPDH was used as a reference gene. Data were acquired using either an ABI Prism 7000 system (Applied Biosystems) or the MX3005p system (Agilent Technologies) and analysed with ABI7000 SDS analysis software or with MxPro QPCR software respectively. Cycle to threshold (C_T_) values were determined for each condition, normalised to GAPDH levels and % mRNA expression normalised to control siRNA calculated as (100/(2 ^ (C_T_ shift)).

## Additional files

Additional file 1: **Figure S1.** Thrombin induces an increase in MLC2 phosphorylation. (A) HUVECs were treated with 1 U/ml thrombin for 10 min, then lysed and analysed by immunoblotting for phospho-MLC2 (pMLC2) and MLC2. GAPDH was used as a loading control. Results for 3 separate experiments (Exp.) are shown. (B) Quantification of pMLC2 levels was performed by individually normalising pMLC2 and MLC2 levels to GAPDH levels. The ratio of the normalised pMLC2 level to the normalised MLC2 level was used as a measure of MLC2 phosphorylation, and is shown for thrombin stimulation as % of control cells (no thrombin). Data are means of 3 independent experiments ± SEM (PDF 166 kb).
