# Supplementary figures and images for: The STRIPAK complex components FAM40A and FAM40B regulate endothelial cell contractility via ROCKs
Source: BMC Cell Biol. 2018 Dec 3;19:26. doi: 10.1186/s12860-018-0175-y (PMC6276190; doi:10.1186/s12860-018-0175-y)

Additional file 1

A

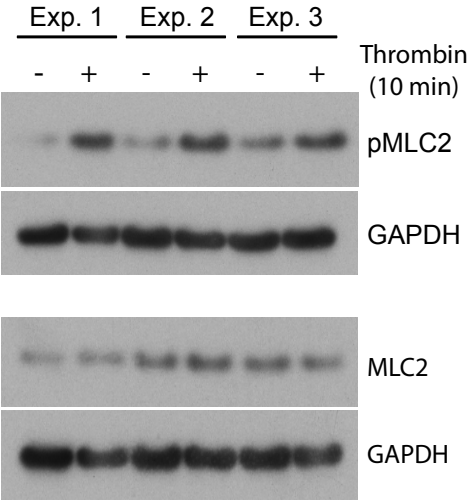

B

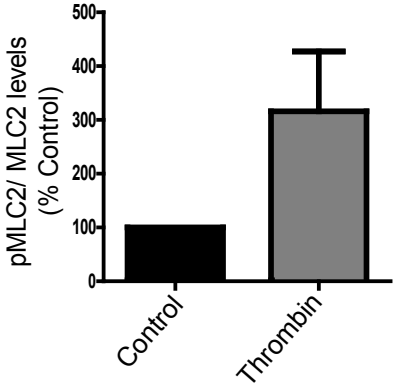

Supplement: Supplementary file 7 — Figure S1. Thrombin induces an increase in MLC2 phosphorylation. (A) HUVECs were treated with 1 U/ml thrombin for 10 min, then lysed and analysed by immunoblotting for phospho-MLC2 (pMLC2) and MLC2. GAPDH was used as a loading control. Results for 3 separate experiments (Exp.) are shown. (B) Quantification of pMLC2 levels was performed by individually normalising pMLC2 and MLC2 levels to GAPDH levels. The ratio of the normalised pMLC2 level to the normalised MLC2 level was used as a measure of MLC2 phosphorylation, and is shown for thrombin stimulation as % of control cells (no thrombin). Data are means of 3 independent experiments ± SEM (PDF 166 kb). [file 12860_2018_175_MOESM1_ESM.pdf]
